# Synthesis and Characterization of Chemically Stable N7-dG Estrone and Catechol Adducts

**DOI:** 10.3390/molecules31101632

**Published:** 2026-05-12

**Authors:** Philip T. Baily, Seongmin Lee

**Affiliations:** The Division of Chemical Biology and Medicinal Chemistry, College of Pharmacy, The University of Texas at Austin, Austin, TX 78712, USA

**Keywords:** estrogen, catechol, quinone, N7-guanine, alkylation, DNA damage, breast cancer, carcinogenesis

## Abstract

Endogenous estrogens are implicated in carcinogenesis through both estrogen receptor-mediated cell proliferation and the direct genotoxicity of reactive metabolites. Oxidative metabolism of estrogens produces catechol estrogens that are further converted to electrophilic *ortho*-quinones capable of alkylating DNA. The prevailing model of mutagenesis proposes that these N3Ade and N7Gua adducts depurinate to form abasic sites that induce mutations initiating hormone-related cancers. However, the mutation spectrum observed in experimental data is inconsistent with this mechanism, and synthetic studies of estrogen-DNA adducts have relied on acidic conditions that artificially promote depurination, leaving stable N7-dG lesions poorly understood. To address this, we synthesized stable N7-dG catechol and estrone adducts using 2′-fluorinated deoxyguanosine, a modification that inhibits N-glycosidic bond cleavage. ROESY 2D NMR spectroscopy revealed through-space correlations consistent with a preferred *anti*-conformation in solution, supported by molecular modeling. Structural analysis suggests that these cationic aryl adducts likely preserve the Watson–Crick base pairing edge but may promote tautomerization capable of altering base pairing and generating G-to-A mutations. These findings provide the first synthesized stable models of N7-dG estrogen adducts and may support an alternative mechanism of estrogen-induced mutagenesis independent of depurination, enabling future biochemical investigations of related DNA repair and mutagenesis.

## 1. Introduction

**Figure 1:**
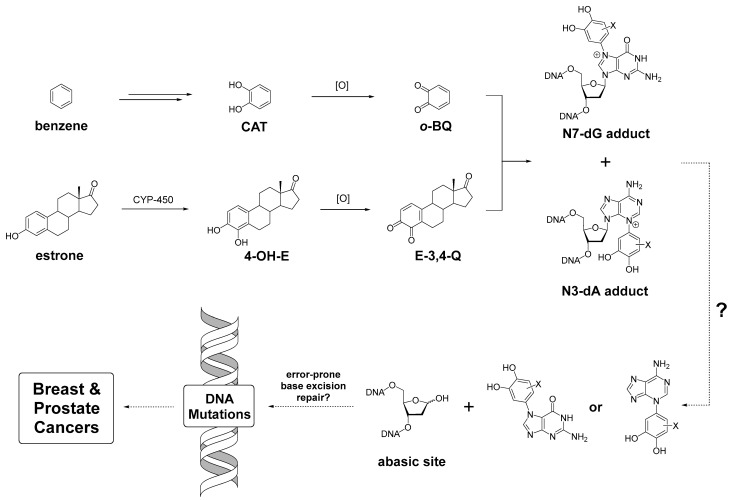
Currently understood pathway of cancer induction via direct DNA damage by *o*-quinone metabolites of benzene and estrone. (CAT = catechol, *o*-BQ = *ortho*-benzoquinone, 4-OH-E = 4-hydroxyestrone, E-3,4-Q = estrone-3,4-quinone).

Endogenous estrogens have been proposed to possess multiple roles in the induction of cancer, including stimulating proliferation of cells by estrogen receptor-mediated signaling, as well as directly damaging DNA [1,2,3,4,5]. Estrogen’s DNA damaging effects stem from the generation of catechol estrogen metabolites by human CYP-450 liver enzymes, which can be further oxidized to highly electrophilic *ortho*-quinones that covalently bind to DNA [1,2,3,5,6,7]. The alkylation of DNA to form predominantly depurinating N3Ade and N7Gua adducts has been reported with estrogen quinones along with other quinone metabolites of compounds including the neurotransmitter dopamine [4,8], the selective estrogen receptor modulator (SERM) lasofoxifene [9], and known carcinogens diethylstilbestrol and benzene [1,2,10]. Conventional knowledge has described the mechanism of carcinogenesis by these lesions to be caused by the generation of abasic sites formed after depurination of N7Gua and N3Ade adducts which leads to mutations initiating breast, prostate, and other human cancers (Figure 1) [2,3,4,5]. Estrogen-DNA adduct ratios have been associated with cancer in both men and women, thus detection of these adducts has potential as a biomarker for breast and prostate cancer risk [11,12].

The first investigations into the direct genotoxicity of estrogens were done by reacting estrogen quinones with deoxyribonucleosides [6]. It was found that the major products of these reactions when treated with estrogen-3,4-quinones (E-3,4-Q) were depurinated N7Gua, whereas estrogen-2,3-quinone (E-2,3-Q) treatment resulted in stable N6-dA and N2-dG adducts [6]. A number of analogous reactions have since been reported with other *o*-quinone metabolites of various compounds with dG and dA in DNA to yield depurinating N7Gua and N3Ade adducts, leading to a hypothesized unified mechanism of *o*-quinone metabolites as carcinogens [4]. These previous synthetic approaches to form N7Gua and N3Ade catechol adducts all used acidic conditions to achieve the Michael addition of nucleobases with the respective *o*-quinones, but these low-pH conditions are also known to promote depurination which may not be occurring in vivo at physiological pH [13,14].

Terashima et al. incorporated synthetic N2-dG and N6-dG-2,3-quinone adducts into oligonucleotides and demonstrated their mutagenicity during DNA replication, producing mainly G-to-T and A-to-T mutations [15,16]. However, E-3,4-Q is more reactive with DNA than E-2,3-Q and this correlates with the mutagenicity of their catechol estrogen precursors 4-OH-E and 2-OH-E [17]. In one study, levels of the catechol estrogen 4-OH-E were nearly four times higher in women with breast carcinoma than without, and this catechol is likely the major carcinogenic metabolite of estrogens [2]. When the human breast epithelial cell line MCF-10F was treated with 4-OH-E, depurinating N3Ade and N7Gua adducts were detected [18]. In mouse and rat tissues, E-3,4-quinone treatment induces predominantly A-to-G but also G-to-A mutations [19,20].

If this mutation spectrum was the result of depurination it would require the random misincorporation of a dG at the abasic site, forming a G:T mispair which is then repaired by BER. However, studies on abasic sites suggest that several DNA polymerases preferentially incorporate dA opposite an abasic site [21,22,23], which is inconsistent with the currently proposed mechanism. In addition, all positively charged N7G and N3A adducts would be expected to lead to a similar mutation spectrum if depurination were the basis for this mutagenicity. An aryl adduct such as catechol or estrone could be expected to help stabilize the positive charge compared to a simple alkyl lesion such as N7MeG. Our laboratory has previously shown N7MeG to be nonmutagenic, forming three hydrogen bonds with dC that adopt canonical Watson–Crick base pairing geometry [24]. However, the ring-opened lesion Me-FapyG is significantly more mutagenic than N7MeG [24]. Depurination of N7-alkyl-dG is also known to be slower in dsDNA compared to at the level of nucleosides [25]. These observations led us to wonder if an alternative mechanism of mutagenicity could be plausible where N7G aryl adducts with stable N-glycosidic bonds can tautomerize to alter base pairing properties and lead to mutations.

Due to the challenges of synthesizing stable N7dG and N3dA estrogen adducts at the nucleoside level without subsequent glycosidic bond cleavage, the downstream processing of these N7Gua and N3Ade lesions by DNA repair enzymes in vitro and in vivo have remained unstudied. While N3-dA estrogen adducts are rapidly depurinating—becoming completely lost from DNA within 1 h [26,27]—the corresponding N7-dG adducts exhibit considerably greater stability, with reported half-lives of about 6 h at 37 °C and pH 4 [20,26,27]. At physiological (natural) pH, the half-life of N7-dG estrogen adducts is expected to be even longer. The bulky N7-dG estrogen adducts may be recognized and removed by DNA repair pathways, including nucleotide excision repair (NER). If the adducts are left unrepaired, replication machinery may bypass these lesions through the activity of error-prone translesion synthesis (TLS) polymerases, potentially leading to mutations. TLS polymerases including Pol η and Pol ζ have significantly more spacious active sites compared to replicative DNA polymerases, allowing them to accommodate bulky lesions and bypass them. DNA crosslinked by oxaliplatin or containing large N7-alkyl adducts, including nitrogen half-mustards and aflatoxin B_1_, have all been shown to undergo bypass by TLS polymerases [28,29,30].

To address the possibility of mutagenicity related to the longer-lived N7dG estrogen adducts, we set out to synthesize a stable model compound that could be used to investigate the consequences of these adducts undergoing DNA repair prior to depurination.

**Figure 2:**
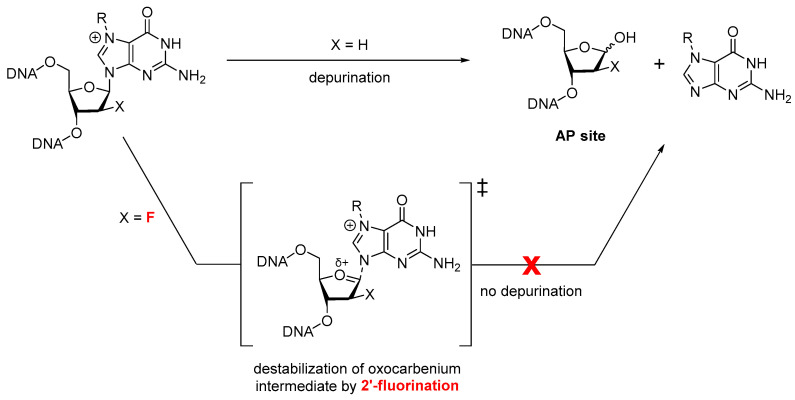
Inhibition of glycosidic bond cleavage via incorporation of a 2′-fluoro group to destabilize the transition state of spontaneous depurination of N7-alkylguanosine adducts.

The mechanism of estrogen-induced DNA damage was investigated by synthesizing *o*-quinone catechol and estrone metabolites and characterizing their reactions with 2′-fluorinated dG, which our laboratory has previously used to stabilize positively charged N7-dG adducts and prevent depurination (Figure 2) [24,31]. We herein report the synthesis of novel stable N7-dG catechol and estrone adducts using 2′-fluoro technology. This study represents first steps toward an alternate mechanism of mutagenicity caused by catechol estrogen quinones and improves our understanding of the effects of estrogens on DNA to inform future biochemical study of the cellular repair and mutagenicity of this damage.

## 2. Results and Discussion

### 2.1. Synthesis of 2′-F-N7-Catechol and Estrone-dG

Previous literature describes the synthesis of depurinated N7Gua catechol and estrone adducts by preparing the respective quinone metabolites using Ag_2_O or MnO_2_ as oxidizing agents in either DMF or MeCN and filtering them directly into a solution of dG in H_2_O/AcOH [1,6]. The acidic pH of these reactions is important because acid catalysis assists the necessary 1,4-Michael addition by providing a proton source to restore aromaticity of the catechol ring. However, low pH can also promote N-glycosidic bond cleavage of nucleosides [13] and is not consistent with the physiological pH where these adducts may be expected to form in vivo.

Using similar methods of preparing *o*-BQ and E-3,4-Q (the respective quinone metabolites of benzene and estrone) in situ, we took the approach of using a 2′-fluorinated deoxyguanosine (2′F-dG) to demonstrate that a stable cationic adduct could be formed without subsequent N-glycosidic bond cleavage (Figure 2). This fluorine modification enhances the chemical stability of the glycosidic bond by destabilizing the transition state of spontaneous depurination, without impacting the normal C2′ endo sugar pucker seen in B-DNA [24,32]. 2′F-dG was synthesized from commercially available starting materials in four steps as previously reported [32]. The catechol estrogen 4-OH-E was prepared from the oxidation of estrone with 2-iodoxybenzoic acid (IBX) [33], and the desired catechol estrone, 4-OH-E, was isolated using silica gel treated with ascorbic acid prior to chromatography in order to prevent oxidative decomposition [34]. IBX was freshly prepared by the protocol of Frigerio et al. [35].

**Figure 3:**
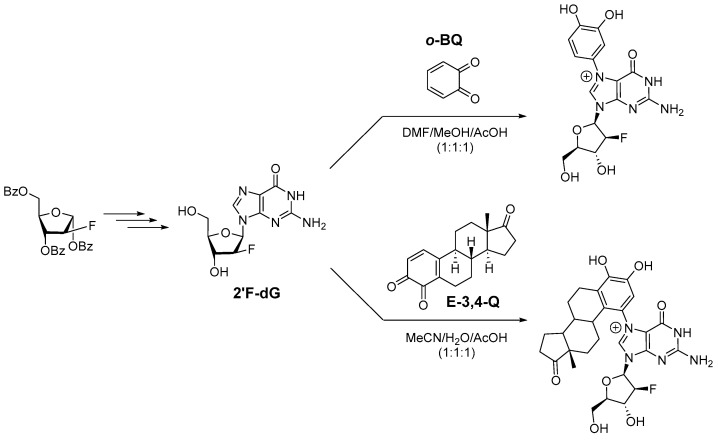
Preparation of 2′-fluoro-2′-deoxyguanosine (2′F-dG) and subsequent N7-alkylation with quinone metabolites to form stable catechol and estrone adducts.

**Figure 4:**
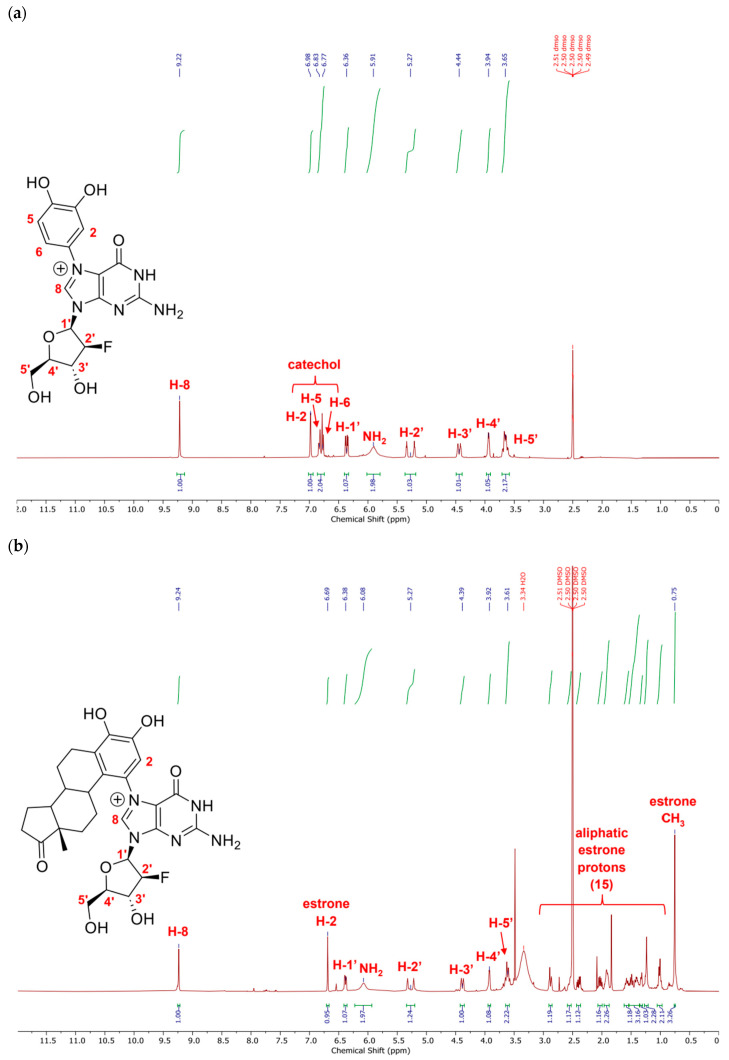
^1^H NMR spectra in DMSO-*d6* of synthesized adducts with peak assignments and integrations. (**a**) 2′-F-N7-catechol dG; (**b**) 2′-F-N7-estrone dG.

Initially, we tested the 1,4-Michael addition reaction in DMF alone and DMF with catalytic amounts of AcOH, but in both of these cases the reaction did not proceed. The addition of H_2_O as a co-solvent led to the formation of the desired product by TLC and ESI-MS, and it was further determined that a polar protic solvent was required for the reaction to take place. We determined that the optimal conditions for synthesis 2′-fluoro-N7-catechol-dG was to run the reaction in DMF/MeOH/AcOH 1:1:1, while MeCN/H_2_O/AcOH 1:1:1 gave the best yields of 2′-fluoro-N7-estrone-dG (Figure 3). As we hypothesized, the products formed from these reactions retained the deoxyribose sugar moiety indicating that fluorination allowed the N-glycosidic bond to withstand the necessary acidic conditions for adduct formation. Both 2′-fluoro-N7-catechol-dG and 2′-fluoro-N7-estrone-dG were successfully isolated and characterized as stable cationic compounds (Figure 4a,b).

Although the isolated yields of 2′-fluoro-N7-catechol-dG and 2′-fluoro-N7-estrone-dG were quite modest (24% and 9.2% respectively), it is worth noting that much of the yield was lost during the purification. By TLC and crude NMR analysis, as much as 40% desired product formation occurred, so access to better purification methods (e.g., preparative HPLC) would likely increase the yield significantly. In addition, there are no major off-target nucleoside products formed by the reaction. Over time, the *o*-quinone reactants simply degrade before allowing full conversion of the 2′F-dG starting material. These results were consistent over several separate reaction trials, and it was found that using more equivalents of *o*-quinone or adding *o*-quinone to the reaction mixture multiple times in batches led to diminishing returns that were not as practical or efficient. The unreacted 2′F-dG starting material can be recovered after the reaction to be used in future syntheses. Given all this, we believe that this synthetic method is able to adequately generate enough product to perform necessary downstream biological studies.

The stability of N7-aryl-2′-F-dG adducts under physiological conditions was evaluated by UV–Vis spectroscopy following incubation in PBS (pH 7.4) at 37 °C for 48 h (see Supporting Information). N7-catechol-2′-F-dG exhibited an absorption maximum at 275 nm that remained unchanged through 24 h, with only minor decreases in absorbance observed at later time points (7% at 24 h and 14% at 48 h; Figure S1), consistent with a half-life beyond 48 h. N7-estrone-2′-F-dG showed even greater stability, with the absorption maximum at 247 nm displaying less than a 5% change in peak intensity after 48 h (Figure S2). These results indicate that both adducts are highly stable under physiological conditions over the timescale examined.

### 2.2. Modeling of Catechol and Estrone dG Adducts Indicates Preference for Anti-Conformer over syn

**Figure 5:**
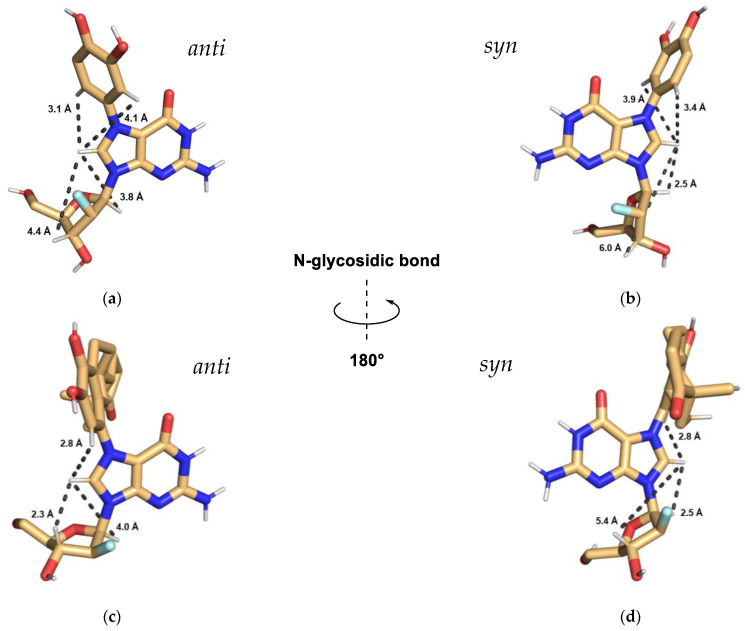
The three-dimensional structures of 2′-fluorinated-N7-catechol and N7-estrone deoxyguanosine adducts modeled in *anti* and *syn* conformations. (**a**) *Anti*-conformer of 2′-fluoro-N7-catechol-dG; (**b**) *syn*-conformer of 2′-fluoro-N7-catechol-dG. (**c**) *Anti*-conformer of 2′-fluoro-N7-estrone-dG; (**d**) *syn*-conformer of 2′-fluoro-N7-estrone-dG.

The three-dimensional structures of 2′-fluoro-N7-catechol-dG and 2′-fluoro-N7-estrone-dG were constructed using Maestro version 14.6 (Schrodinger, LLC, New York, NY, USA) and energy minimized in both *anti* and *syn* conformations (Figure 5). For both catechol and estrone adducts, the structures are consistent with *anti*-conformer being preferred in solution based on 2D NMR (ROESY) analysis. ROESY has been used for conformational analysis of a variety of compounds including macrolides, peptides, and nucleotides [36,37,38,39]. ROESY cross-peaks are used to identify through-space correlations of protons within a chemical structure, and typically detect correlations of protons within 5Å of each other [38,39]. This information can be used alongside molecular modeling to interpret the predominant conformation in solution.

**Figure 6:**
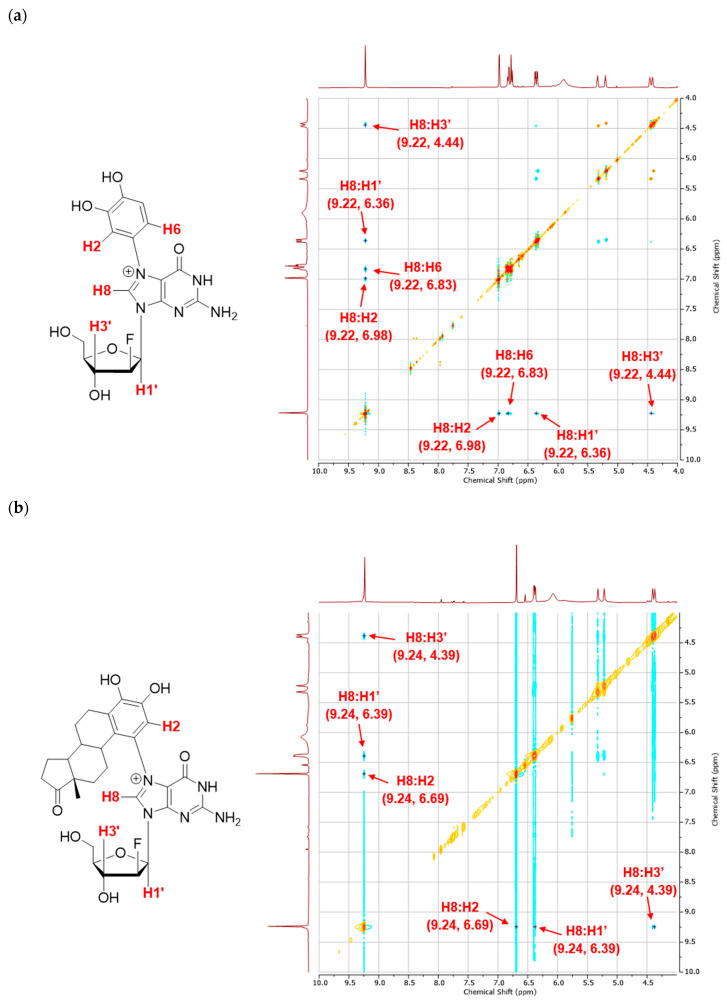
ROESY 2D NMR spectra in DMSO-*d6* with selected cross-peaks confirming N7-alkylation and indicative of a preference for the *anti*-conformation over *syn* in solution: (**a**) 2′-F-N7-catechol dG; (**b**) 2′-F-N7-estrone dG.

ROE cross-peaks were observed between the C-8 proton of the guanine moiety with the adjacent aromatic protons in both the catechol and estrone rings (Figure 6a,b). If alkylation took place at another site instead of N7, no correlation between these protons would have been observed, which confirms the regioselectivity of adduct formation. Additionally, ROESY cross-peaks were seen between the guanine C-8 proton and nearby deoxyribose 1′ and 3′ protons (Figure 6a,b). In particular, the correlation of the C-8 proton with the 3′-H gives insight into the preferred conformation of the two N7G adducts in solution. As shown in Figure 5, when these structures are modeled in the *anti*-conformation, the distances between the C8 and 3′ protons are well within 5Å, whereas the *syn*-conformers place these protons at distances of 5.4 Å and 6.0 Å where ROESY cross-peaks would be highly unlikely to be detected.

**Figure 7:**
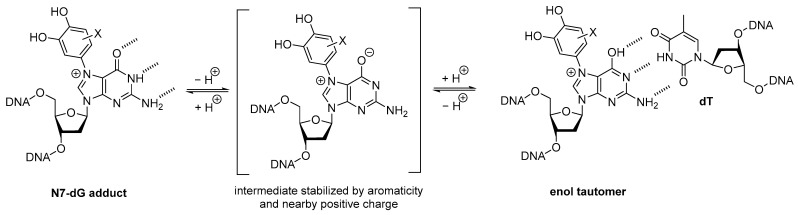
Potential mechanism of tautomerization that could lead to altered base pairing and misrecognition by DNA repair enzymes, leading to G-to-A mutations.

Notably, the *anti*-conformer would preserve the base pairing face in the same orientation as standard dG in B-DNA. This observation could indicate that further modification such as tautomerization to an enol form that allows altered base pairing and misrecognition by DNA repair enzymes is required to explain mutagenicity of this lesion. As shown in Figure 7, it is possible that the presence of the aryl moiety and positive charge at the N7-position could facilitate the formation of the enol tautomer of guanine by stabilizing the enolate intermediate. The resulting enol tautomer would possess a base pairing face reminiscent of the hydrogen bond donors and acceptor of 2,6-diaminopurine that is known to form three stable hydrogen bonds with thymine in the opposite DNA strand. Incorporation of dT opposite to this lesion by DNA polymerases would lead to the G-to-A mutations that have been reported in the literature [19,20]. This hypothesis is consistent with previous structural data showing that N7MeG alters the hydrogen bonding patterns of guanine in dsDNA via enol tautomerization [40,41]. A major limitation of this study is that the ROESY NMR was conducted in DMSO-*d6* rather than physiological conditions, pH, and dsDNA environment, which would be needed to more fully assess whether the anti-conformation predominates in vivo. While our proposed tautomerization mechanism appears consistent with these data, it should be considered as a working hypothesis that would benefit from further investigation to evaluate its accuracy. Wobble base pairing could be an alternative explanation, and X-ray crystallography of a polymerase in complex with dsDNA containing N7-catechol/estrone-2′F-dG lesions could help differentiate between these possibilities.

## 3. Materials and Methods

Caution: catechol and estrone quinones are toxic and suspected human carcinogens, requiring handling according to NIH guidelines [42].

General methods

Reagents, such as catechol, estrone, and activated MnO_2_ were purchased from Thermo Fisher Scientific Inc. (Fair Lawn, NJ, USA), and used as received. Unless otherwise indicated, all reactions were carried out under a positive pressure of argon in anhydrous solvents and the reaction flasks were fitted with rubber septa for the introduction of substrates and reagents via syringe. Progress of reactions was monitored by thin-layer chromatography (TLC) in comparison with the starting materials. All TLC analyses were carried out on Silica Gel 60 F254 TLC plates (EMD Chemicals Inc., Darmstadt, Germany), thickness of 0.25 mm. The plates were visualized by ultraviolet illumination at 254 nm and immersion in visualizing solution. The two commonly employed TLC visualizing solutions were: (i) *p*-anisaldehyde solution (1350 mL absolute ethanol, 50 mL concentrated H_2_SO_4_, 37 mL *p*-anisaldehyde) and I_2_ chamber. ^1^H NMR and ^13^C NMR spectra were recorded on a Varian Mercury 400 (400 MHz; Palo Alto, CA, USA) instrument.

Synthesis of 2′-fluoro-N7-catechol-dG

To an oven-dried vial was added CAT (550 mg, 5.0 mmol, 10 Eq), followed by anhydrous DMF (5.0 mL) at 0 °C in an ice bath. To this stirring solution, Ag_2_O (2.32 g, 10.0 mmol, 20 Eq) was added and the resulting suspension was stirred for 30 min at 0 °C. The dark red-orange suspension was centrifuged, then quinone-containing supernatant was added directly to a round bottom flask containing a stirring solution of 2′F-dG (143 mg, 0.50 mmol, 1 Eq) in AcOH/MeOH 1:1 (10.0 mL) through 0.2 µm nylon syringe filter at ambient temperature. The reaction stirred for 48 h and was monitored by TLC until *o*-BQ had been completely consumed. The mixture was poured into 100 mL diethyl ether (Et_2_O), resulting in the formation of 175 mg of crude precipitates containing both unreacted 2′F-dG and desired product which were collected by filtration. This mixture was then subjected to silica gel column chromatography using a gradient of 10–25% MeOH in CH_2_Cl_2_ to elute all impurities and recollect unreacted 2′F-dG, then silica gel was dried of solvent and resuspended in 100 mL of MeOH. After stirring for 2 h, the supernatant was filtered and concentrated to afford 45 mg (24%) of pure 2′-fluoro-N7-catechol-dG.

^1^H NMR (400 MHz, DMSO-*d*_6_) δ 9.22 (s, 1H, H-8), 6.98 (s, 1H, catechol H-2), 6.83 (d, *J* = 8.4 Hz, 1H, catechol H-5), 6.77 (d, *J* = 8.4 Hz, 1H, catechol H-6), 6.36 (dd, *J* = 13.3, 4.2 Hz, 1H, H-1′), 5.91 (br s, 2H, NH_2_), 5.27 (dt, *J* = 52.0, 3.9 Hz, 1H, H-2′), 4.44 (dt, *J* = 17.6, 4.3 Hz, 1H, H-3′), 3.94 (q, *J* = 4.7 Hz, 1H, H-4′), 3.65 (qd, *J* = 12.3, 4.4 Hz, 2H, H-5′).

^13^C NMR (126 MHz, DMSO-*d*_6_) δ 163.66, 161.93, 150.29, 146.66, 132.30, 126.11, 116.23, 115.09, 113.61, 108.39, 96.00, 94.08, 85.38, 83.43, 83.26, 72.60, 72.37, 60.59.

HR-ESI-MS: Calculated for C_16_H_17_FN_5_O_6_^+^ *m*/*z* [M]^+^ = 394.1157; Observed *m*/*z* [M]^+^ = 394.1165.

Synthesis of 2′-fluoro-N7-estrone-dG

To an oven-dried vial was added 4-OH-E (572 mg, 2.0 mmol, 5 Eq), followed by anhydrous MeCN (6.6 mL) at 0 °C in an ice bath. To this stirring solution, activated MnO_2_ (1.74 g, 20.0 mmol, 50 Eq) was added and the resulting suspension was stirred for 30 min at 0 °C. The dark red-orange suspension was centrifuged, then quinone-containing supernatant was added directly to a round bottom flask containing a stirring solution of 2′F-dG (114 mg, 0.40 mmol, 1 Eq) in AcOH/H_2_O 1:1 (13.4 mL) through 0.2 µm nylon syringe filter at ambient temperature. The reaction stirred for 48 h and was monitored by TLC until E-3,4-Q had been completely consumed. The mixture was extracted with CH_2_Cl_2_ to remove estrone byproducts, while unreacted 2′F-dG and desired product remained in the aqueous layer which was evaporated to yield 160 mg of crude mixture. This mixture was then subjected to silica gel column chromatography using a gradient of 10–25% MeOH in EtOAc to elute all impurities and recollect unreacted 2′F-dG, then the silica gel was dried of solvent and resuspended in 100 mL of MeOH. After stirring for 2 h, the supernatant was filtered and concentrated to afford 21 mg (9.2%) of pure 2′-fluoro-N7-estrone-dG.

^1^H NMR (400 MHz, DMSO-*d*_6_) δ 9.24 (s, 1H, H-8), 6.69 (s, 1H, estrone H-2), 6.38 (dd, *J* = 11.8, 4.4 Hz, 1H, H-1′), 6.07 (br s, 2H, NH_2_), 5.27 (dt, *J* = 52.3, 4.2 Hz, 1H, H-2′), 4.39 (dt, *J* = 18.0, 4.6 Hz, 1H, H-3′), 3.92 (q, *J* = 4.7 Hz, 1H, H-4′), 3.61 (dd, *J* = 12.5, 5.7 Hz, 2H, H-5′), 2.91–2.85 (m, 1H), 2.56 (d, *J* = 13.6 Hz, 1H), 2.40 (dd, *J* = 19.2, 8.9 Hz, 1H), 2.02 (dd, *J* = 18.7, 9.2 Hz, 1H), 1.92 (tt, *J* = 13.3, 6.0 Hz, 2H), 1.58 (dd, *J* = 15.9, 7.3 Hz, 1H), 1.54–1.36 (m, 3H), 1.32 (d, *J* = 8.3 Hz, 1H), 1.25 (d, *J* = 11.6 Hz, 2H), 1.01 (d, *J* = 9.4 Hz, 2H), 0.75 (s, 3H, estrone CH_3_).

^13^C NMR (126 MHz, DMSO-*d*_6_) δ 167.71, 162.44, 149.49, 144.19, 142.39, 132.49, 125.79, 125.30, 124.51, 113.70, 107.59, 95.09, 93.54, 84.69, 82.73, 82.59, 71.83, 71.64, 59.89, 49.72, 49.53, 47.25, 43.38, 35.18, 31.52, 30.70, 25.25, 24.37, 24.06, 21.16, 13.72.

HR-ESI-MS: Calculated for C_28_H_33_FN_5_O_7_^+^ *m*/*z* [M]^+^ = 570.2359; Observed *m*/*z* [M]^+^ = 570.2363.

## 4. Conclusions

Taken together, our findings address a gap in the current model of estrogen-induced mutagenesis. While the current paradigm attributes the genotoxicity of catechol estrogen quinones primarily to depurination of N7-dG and N3-dA adducts, the lack of stable models has limited direct investigation of the mutagenic properties of N7-dG lesions prior to N-glycosidic bond cleavage. Using the methods described in this study, stable N7-dG catechol and estrogen adducts can be synthesized while avoiding depurination. Our 2D NMR and structural analysis suggests that these lesions could retain the Watson–Crick base pairing face and *anti*-conformation in solution. We hypothesize that the presence of positively charged N7-aryl adducts may promote tautomerization capable of altering base pairing, offering a plausible explanation for the G-to-A mutations observed in previous experiments. These results represent a preliminary step in future work that may help support the notion that estrogen quinone-induced mutagenesis could occur through mechanisms independent of depurination and may help establish a foundation for future biochemical studies examining the repair and biological consequences of stable N7-guanine estrogen adducts. A better understanding of the underlying mechanisms of mutagenesis caused by toxic estrogen metabolites would contribute to prevent or treat cancers associated with these pathways.

## Data Availability

All supporting data are included within the main article.

## Supplementary Materials

The following supporting information can be downloaded at: https://www.mdpi.com/article/10.3390/molecules31101632/s1. File S1. UV-Vis stability testing; Figure S1. Stacked UV-Vis data of N7-CAT-2′-F-dG; Figure S2. Stacked UV-Vis data of N7-etrone-2′-F-dG; Table S1. Raw data for Figure S1; Table S2. Raw data for Figure S2.

## Abbreviations

The following abbreviations are used in this manuscript:

AcOH	Acetic acid
BER	Base excision repair
CAT	Catechol
CYP-450	Cytochrome P-450
DMF	Dimethylformamide
DNA	Deoxyribonucleic acid
dsDNA	Double-stranded DNA
dG	Deoxyguanosine
dA	Deoxyadenosine
E-2,3-Q	Estrogen-2,3-quinone
E-3,4-Q	Estrogen-3,4-quinone
ESI-MS	Electrospray ionization mass spectrometry
IBX	2-iodoxybenzoic acid
MeCN	Acetonitrile
Me-FapyG	N7-methyl-formamidopyrimidine guanine
N3Ade	N3-adenine
N6Ade	N6-adenine
N2Gua	N2-guanine
N7Gua	N7-guanine
N7MeG	N7-methylguanine
NER	Nucleotide excision repair
NMR	Nuclear magnetic resonance
*o*-BQ	*Ortho*-benzoquinone
ROESY	Rotating frame Nuclear Overhauser Effect Spectroscopy
SERM	Selective estrogen receptor modulator
TLC	Thin-layer chromatography
TLS	Translesion synthesis
2′F-dG	2′-fluoro-2′-deoxyguanosine
2-OH-E	2-hydroxyestrone
4-OH-E	4-hydroxyestrone
