# Supplementary material for: Synthesis and Characterization of Chemically Stable N7-dG Estrone and Catechol Adducts
Source: Molecules. 2026 May 12;31(10):1632. doi: 10.3390/molecules31101632 (PMC13209890; doi:10.3390/molecules31101632)
Supplement: Supplementary file 1 [file molecules-31-01632-s001.zip › molecules-4228504-supplementary.pdf]

Supplementary Materials File S1.

UV-Vis Stability Testing

Using a ThermoFisher Evolution UV-Vis spectrophotometer, a study was conducted to probe the stability of our N7-aryl-2′-F-dG compounds under physiological conditions. Both N7-catechol-2′F-dG and N7-estrone-2′F-dG were dissolved in aqueous phosphate-buffered saline (PBS) buffer [137 mM NaCl, 2.7mM KCl, 10 mM Na2HPO4, 1.8 mM KH2PO4] at pH 7.4 at a nucleoside concentration of 1.0 mM. An aliquot of each was diluted by a factor of 10 with additional buffer to a final concentration of 100 μM and incubated at 37°C for 48 hours. At set time points, a UV-Vis scan of wavelengths 220-400 nM was performed in a quartz cuvette and the results were compiled. As shown in Figure S1, the UV-Vis curve of N7-catechol-2′F-dG has a maxima at 275 nm that does not shift or decrease in intensity until the 24 hour and 48 hour marks. Even at these time points the overall decrease is slight (a 7% decrease in peak absorbance at 24 hours and 14% decrease at 48 hours), indicating that the half-life of the compound is likely well beyond 48 hours under physiological conditions. As for N7-estrone-2′F-dG, there is an even less noticeable change in the UV-Vis spectrum over time than for the catechol adduct (Figure S2). The absorption maximum at 247 nm at the 48 hour time point is within 5% of the peak absorbance for the N7-estrone adduct.

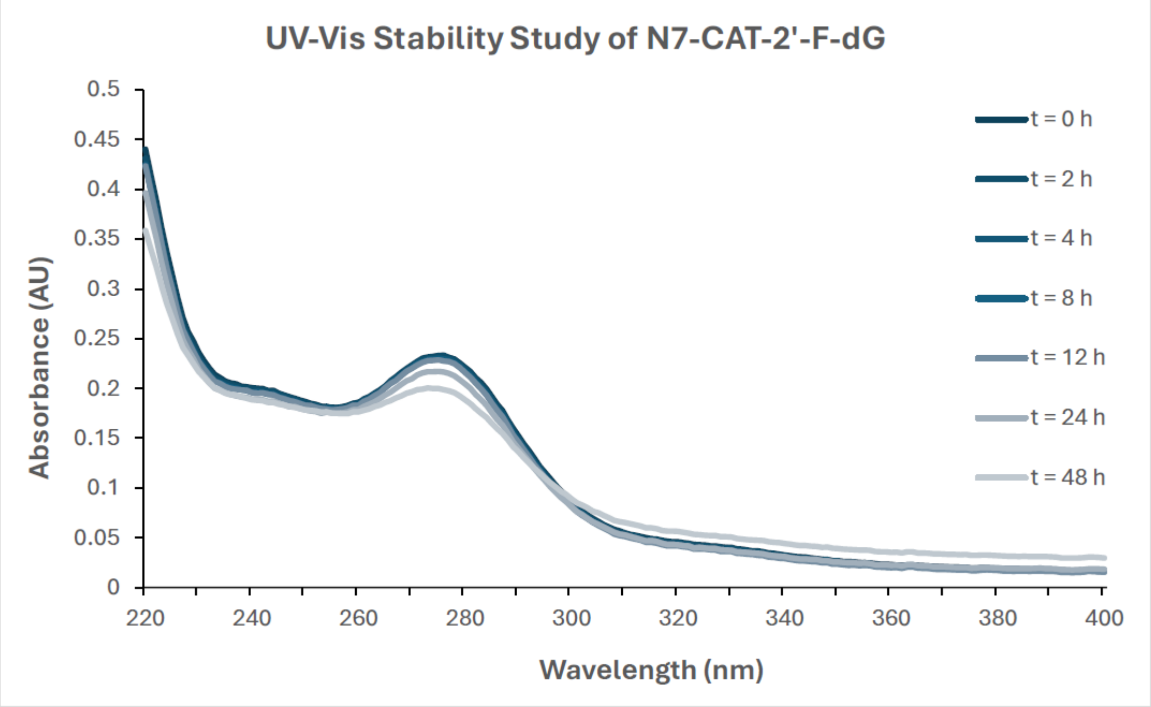

Figure S1. Stacked UV-Vis data of N7-CAT-2′-F-dG incubated at 37°C over a 48 hour period

Table S1. Raw data for Figure S1.

Wavelength (nm)	t = 0 h	t = 2 h	t = 4 h	t = 8 h	t = 12 h	t = 24 h	t = 48 h
220	0.440535	0.431483	0.425743	0.424321	0.423522	0.396555	0.358681
221	0.414227	0.40478	0.399127	0.39855	0.397814	0.372896	0.339639
222	0.389678	0.379527	0.374896	0.374525	0.373015	0.35098	0.322463
223	0.361695	0.351756	0.347243	0.346426	0.345549	0.326484	0.302873
224	0.337107	0.326415	0.322074	0.321291	0.320709	0.304506	0.284988

225	0.314455	0.305866	0.301294	0.300847	0.300411	0.285343	0.270427
226	0.291806	0.283989	0.279405	0.27875	0.277798	0.265217	0.254772
227	0.271573	0.264301	0.26116	0.259919	0.25898	0.249086	0.24112
228	0.257073	0.252674	0.249058	0.248709	0.247841	0.238668	0.233085
229	0.247314	0.24253	0.239404	0.23785	0.237149	0.229599	0.224952
230	0.236137	0.231474	0.228953	0.227066	0.227108	0.219174	0.216765
231	0.227883	0.223825	0.221281	0.220513	0.219475	0.212556	0.211217
232	0.220333	0.217407	0.214202	0.214006	0.212655	0.206036	0.205219
233	0.214261	0.210874	0.207696	0.207224	0.207145	0.200339	0.200559
234	0.210898	0.208602	0.206009	0.205804	0.205023	0.198218	0.198069
235	0.207328	0.205844	0.203446	0.202844	0.201698	0.195739	0.195081
236	0.205506	0.203946	0.201528	0.200889	0.199863	0.193936	0.193736
237	0.204818	0.202723	0.200665	0.200439	0.199154	0.192426	0.193237
238	0.202359	0.201128	0.198697	0.198788	0.198461	0.19154	0.191268
239	0.201818	0.201131	0.198806	0.198034	0.19828	0.191106	0.190118
240	0.200938	0.199571	0.196792	0.196203	0.195861	0.189336	0.188606
241	0.200177	0.199656	0.197194	0.196829	0.195145	0.189687	0.188201
242	0.199826	0.198891	0.196573	0.195508	0.19558	0.188753	0.187897
243	0.197992	0.197675	0.195076	0.194175	0.194453	0.186811	0.186039
244	0.198059	0.197234	0.195035	0.194001	0.193518	0.187622	0.186076
245	0.19513	0.194827	0.192099	0.191743	0.191166	0.185198	0.184382
246	0.193364	0.192781	0.190637	0.190079	0.189545	0.183763	0.183278
247	0.19174	0.19139	0.188523	0.187686	0.187845	0.181657	0.181784
248	0.190295	0.189269	0.187683	0.187453	0.186647	0.182014	0.181088
249	0.188352	0.18715	0.185177	0.185285	0.184918	0.179797	0.179777
250	0.186874	0.18656	0.183773	0.183353	0.18307	0.178485	0.178266
251	0.185016	0.184161	0.182138	0.182673	0.182792	0.17731	0.177882
252	0.183912	0.183742	0.181721	0.1813	0.180952	0.176806	0.177443
253	0.182195	0.181696	0.180153	0.179682	0.179675	0.175041	0.176198
254	0.181015	0.182182	0.179797	0.180062	0.179153	0.176233	0.176423
255	0.181133	0.180389	0.179208	0.17938	0.178354	0.175605	0.175136
256	0.180711	0.181117	0.178731	0.179315	0.178979	0.175	0.175
257	0.181544	0.181441	0.179664	0.179342	0.179524	0.175572	0.174688
258	0.183054	0.182553	0.180417	0.180277	0.180785	0.176404	0.17474
259	0.184895	0.185129	0.183434	0.183243	0.182919	0.178527	0.176387
260	0.186205	0.185328	0.183428	0.183689	0.183136	0.178836	0.176559
261	0.188164	0.189454	0.187118	0.187467	0.186785	0.181919	0.178146
262	0.190524	0.191834	0.190269	0.189955	0.189234	0.184101	0.179618
263	0.194028	0.1956	0.193927	0.192966	0.192737	0.186524	0.181988
264	0.197657	0.198788	0.196466	0.196261	0.195891	0.189761	0.183346
265	0.202457	0.202676	0.201497	0.200964	0.200904	0.193518	0.186018
266	0.206776	0.207965	0.205638	0.205244	0.20501	0.196534	0.187925
267	0.210378	0.211748	0.209801	0.209258	0.208107	0.200274	0.190622
268	0.214751	0.216153	0.213325	0.213195	0.212784	0.204027	0.192584
269	0.218457	0.220268	0.217951	0.217414	0.217285	0.207607	0.195286
270	0.222655	0.223662	0.221451	0.220827	0.220257	0.209627	0.19669
271	0.226087	0.22728	0.225227	0.224809	0.223474	0.213524	0.198643
272	0.227601	0.230588	0.227166	0.226972	0.22619	0.215691	0.199445
273	0.230628	0.231835	0.229311	0.229094	0.227823	0.216847	0.200664
274	0.232066	0.232403	0.230017	0.229364	0.228145	0.216874	0.200074
275	0.232548	0.23306	0.230898	0.229566	0.228784	0.217126	0.19987

276	0.232072	0.233483	0.22994	0.229244	0.227706	0.216576	0.19869
277	0.231061	0.231228	0.229228	0.227704	0.227044	0.21515	0.197663
278	0.229242	0.229545	0.227104	0.22591	0.22485	0.2126	0.195821
279	0.225853	0.225199	0.223326	0.221373	0.22099	0.208896	0.192519
280	0.221301	0.220994	0.217817	0.21718	0.216059	0.204608	0.188685
281	0.217036	0.216593	0.213705	0.212456	0.211374	0.200159	0.184767
282	0.210822	0.209903	0.207216	0.206618	0.205312	0.193909	0.17981
283	0.205769	0.203518	0.201775	0.200378	0.19934	0.188626	0.175136
284	0.199636	0.19794	0.195501	0.193725	0.193092	0.183041	0.170844
285	0.191841	0.189645	0.186985	0.185781	0.185197	0.1757	0.164821
286	0.184204	0.182198	0.179351	0.178424	0.177931	0.168775	0.159505
287	0.177929	0.176034	0.173923	0.172462	0.171563	0.163305	0.154802
288	0.168397	0.167423	0.16502	0.16407	0.162561	0.155116	0.148685
289	0.160281	0.157591	0.155141	0.154061	0.153034	0.146298	0.141559
290	0.152414	0.150249	0.148099	0.146756	0.146189	0.139866	0.136452
291	0.144525	0.142298	0.140496	0.139555	0.138615	0.133097	0.13064
292	0.137626	0.135192	0.133308	0.132218	0.131158	0.126628	0.125278
293	0.13002	0.127108	0.125144	0.124595	0.123463	0.119418	0.119801
294	0.122005	0.120613	0.118693	0.117837	0.116966	0.113357	0.115224
295	0.115625	0.113664	0.11231	0.111362	0.110531	0.107292	0.110065
296	0.109341	0.10714	0.105058	0.104448	0.103726	0.101136	0.105255
297	0.102818	0.101093	0.099152	0.098008	0.09755	0.095749	0.101117
298	0.097411	0.095267	0.093158	0.092371	0.091486	0.090367	0.096628
299	0.091839	0.090358	0.088607	0.087805	0.087153	0.086074	0.09345
300	0.086482	0.084456	0.083079	0.08206	0.081336	0.081105	0.08897
301	0.081062	0.079522	0.077639	0.077181	0.076304	0.076139	0.085261
302	0.07711	0.076066	0.074318	0.073467	0.072396	0.073256	0.0827
303	0.073013	0.071567	0.070433	0.069418	0.069085	0.069479	0.07996
304	0.069523	0.067819	0.066773	0.065755	0.065179	0.065759	0.076658
305	0.066512	0.065479	0.063725	0.063214	0.062227	0.063569	0.075059
306	0.063263	0.062498	0.061196	0.060548	0.059552	0.060794	0.072476
307	0.060893	0.06017	0.058473	0.057468	0.056958	0.058021	0.069985
308	0.058415	0.056671	0.055441	0.054778	0.053822	0.05538	0.067409
309	0.05703	0.055349	0.053723	0.052842	0.052241	0.053691	0.066239
310	0.054985	0.054561	0.053044	0.05185	0.051179	0.0528	0.065242
311	0.053492	0.052744	0.051537	0.05031	0.049733	0.051269	0.064031
312	0.052633	0.05179	0.0509	0.04918	0.048542	0.049933	0.062927
313	0.05136	0.050466	0.048764	0.048024	0.047303	0.048217	0.061546
314	0.050127	0.048094	0.047241	0.046106	0.045418	0.046764	0.059938
315	0.049046	0.049071	0.047524	0.046709	0.045356	0.047006	0.060019
316	0.048696	0.047736	0.046438	0.04534	0.044386	0.046208	0.058972
317	0.04749	0.046576	0.044887	0.043854	0.043125	0.044546	0.057366
318	0.04655	0.04499	0.043891	0.042595	0.041749	0.043351	0.056623
319	0.045937	0.04533	0.044023	0.042573	0.041803	0.043314	0.056532
320	0.045983	0.045242	0.04411	0.042885	0.041657	0.043014	0.05623
321	0.045098	0.044274	0.042789	0.041514	0.040789	0.042138	0.05531
322	0.044337	0.043956	0.04258	0.041245	0.040359	0.041702	0.05468
323	0.043429	0.041953	0.040641	0.039649	0.038624	0.039867	0.053285
324	0.042971	0.041959	0.040471	0.039529	0.038541	0.040179	0.053116
325	0.042578	0.041875	0.040421	0.039088	0.038425	0.039684	0.052608
326	0.041816	0.041351	0.039744	0.038867	0.037827	0.039087	0.05209

327	0.041784	0.041208	0.039843	0.038509	0.037758	0.039096	0.052067
328	0.040987	0.040049	0.038571	0.037438	0.036296	0.037801	0.051088
329	0.040438	0.039903	0.03867	0.037587	0.036255	0.037782	0.051063
330	0.040408	0.03985	0.03817	0.036997	0.036064	0.037426	0.050653
331	0.038921	0.038058	0.036932	0.035538	0.034503	0.035938	0.0493
332	0.038625	0.037776	0.036375	0.035067	0.033974	0.035527	0.04861
333	0.037646	0.037054	0.035767	0.034035	0.033486	0.035248	0.048052
334	0.03689	0.036295	0.035406	0.033837	0.033214	0.034643	0.047667
335	0.036879	0.035997	0.034537	0.033213	0.032364	0.034282	0.047524
336	0.035881	0.035164	0.034188	0.032789	0.031982	0.03375	0.047098
337	0.034786	0.033916	0.033047	0.031593	0.030543	0.032496	0.04601
338	0.034292	0.033555	0.032295	0.030886	0.030268	0.032107	0.045327
339	0.033499	0.032895	0.031895	0.030419	0.029469	0.031358	0.044933
340	0.03268	0.032166	0.031252	0.03005	0.028998	0.031198	0.044236
341	0.032096	0.031102	0.030534	0.028648	0.028014	0.030039	0.043536
342	0.03111	0.030462	0.029155	0.028121	0.027261	0.02931	0.042767
343	0.03077	0.029779	0.028728	0.027573	0.026595	0.028623	0.042219
344	0.030448	0.029539	0.028641	0.027089	0.02664	0.028806	0.042074
345	0.029692	0.029397	0.028064	0.026944	0.026178	0.028234	0.041591
346	0.02896	0.028838	0.027913	0.026472	0.025902	0.028105	0.041324
347	0.028193	0.028038	0.026621	0.025544	0.024817	0.027094	0.040409
348	0.027858	0.028065	0.026889	0.02546	0.024763	0.027068	0.040491
349	0.027023	0.026652	0.025438	0.02433	0.023376	0.025788	0.039418
350	0.027	0.026379	0.025525	0.023934	0.023445	0.025659	0.039267
351	0.026225	0.02564	0.024566	0.023587	0.022909	0.025305	0.038672
352	0.025984	0.02528	0.02447	0.023167	0.022283	0.02484	0.038142
353	0.025671	0.025206	0.024144	0.023344	0.022524	0.024788	0.038072
354	0.025267	0.024608	0.023556	0.022687	0.021888	0.024394	0.037656
355	0.024749	0.025002	0.024175	0.022886	0.022241	0.024717	0.037531
356	0.024484	0.024473	0.023686	0.022602	0.022055	0.02413	0.037267
357	0.02404	0.023036	0.022073	0.021212	0.020641	0.022901	0.036216
358	0.023409	0.022893	0.022075	0.020994	0.02031	0.022743	0.035914
359	0.02339	0.022672	0.021943	0.020812	0.020005	0.022708	0.035804
360	0.023169	0.022263	0.021207	0.020508	0.01973	0.022131	0.035228
361	0.022608	0.022664	0.021677	0.020817	0.020163	0.022564	0.035531
362	0.022318	0.02159	0.020708	0.019889	0.019276	0.021672	0.034798
363	0.022565	0.022777	0.02216	0.021243	0.020554	0.022926	0.035725
364	0.022505	0.022906	0.022205	0.021165	0.020653	0.023059	0.035856
365	0.022329	0.021996	0.021185	0.020132	0.019525	0.02188	0.034803
366	0.022025	0.021748	0.021113	0.019805	0.019192	0.021622	0.03468
367	0.021741	0.021414	0.020702	0.019612	0.019155	0.021553	0.034511
368	0.021173	0.020986	0.019957	0.018932	0.018452	0.020728	0.033905
369	0.021025	0.020879	0.020069	0.018798	0.018152	0.020756	0.033704
370	0.020856	0.020599	0.019815	0.018543	0.01795	0.020647	0.033467
371	0.020627	0.020348	0.01952	0.01845	0.01811	0.020389	0.033285
372	0.020727	0.02031	0.019487	0.018347	0.017878	0.020278	0.033165
373	0.020302	0.019938	0.019181	0.018046	0.017323	0.020064	0.03292
374	0.020133	0.020196	0.019527	0.018393	0.017891	0.020286	0.033146
375	0.019634	0.019145	0.018484	0.017529	0.016768	0.019383	0.032375
376	0.019596	0.019116	0.018386	0.017728	0.016971	0.019559	0.03227
377	0.01975	0.019716	0.018934	0.018118	0.017569	0.020188	0.032557

378	0.019569	0.019919	0.019105	0.018261	0.017648	0.020261	0.032671
379	0.01938	0.019643	0.019033	0.017856	0.017405	0.019822	0.032369
380	0.019379	0.019316	0.018655	0.017537	0.017112	0.019745	0.032127
381	0.019051	0.019004	0.018339	0.017335	0.016706	0.019271	0.031835
382	0.01879	0.018803	0.018171	0.016987	0.016622	0.018941	0.031444
383	0.018684	0.018638	0.017941	0.016954	0.0162	0.018887	0.031341
384	0.018641	0.018668	0.017876	0.016828	0.016476	0.018975	0.031171
385	0.018393	0.018504	0.0179	0.01687	0.016451	0.019051	0.031373
386	0.01848	0.018413	0.017713	0.016644	0.016255	0.018994	0.031092
387	0.018102	0.018558	0.018036	0.017024	0.016491	0.019191	0.031245
388	0.018211	0.018802	0.017896	0.016923	0.016553	0.019404	0.031174
389	0.01802	0.01835	0.017992	0.016933	0.01641	0.019005	0.031095
390	0.01799	0.018303	0.017787	0.016811	0.016428	0.019018	0.030906
391	0.017854	0.017926	0.017341	0.016529	0.015905	0.018745	0.030662
392	0.01767	0.017005	0.016264	0.015579	0.015143	0.01771	0.029663
393	0.017497	0.017044	0.016375	0.015534	0.014979	0.017898	0.029664
394	0.017247	0.016979	0.016105	0.015382	0.014965	0.017705	0.029737
395	0.017342	0.017278	0.016372	0.015431	0.01512	0.017746	0.02965
396	0.017246	0.017544	0.016796	0.015988	0.015517	0.018199	0.030076
397	0.017373	0.018169	0.017542	0.016546	0.016179	0.018611	0.03061
398	0.017284	0.018082	0.01742	0.016474	0.016041	0.018747	0.030401
399	0.017156	0.017819	0.017099	0.016131	0.015821	0.018649	0.030087
400	0.01719	0.017482	0.01663	0.015791	0.015316	0.018228	0.02971

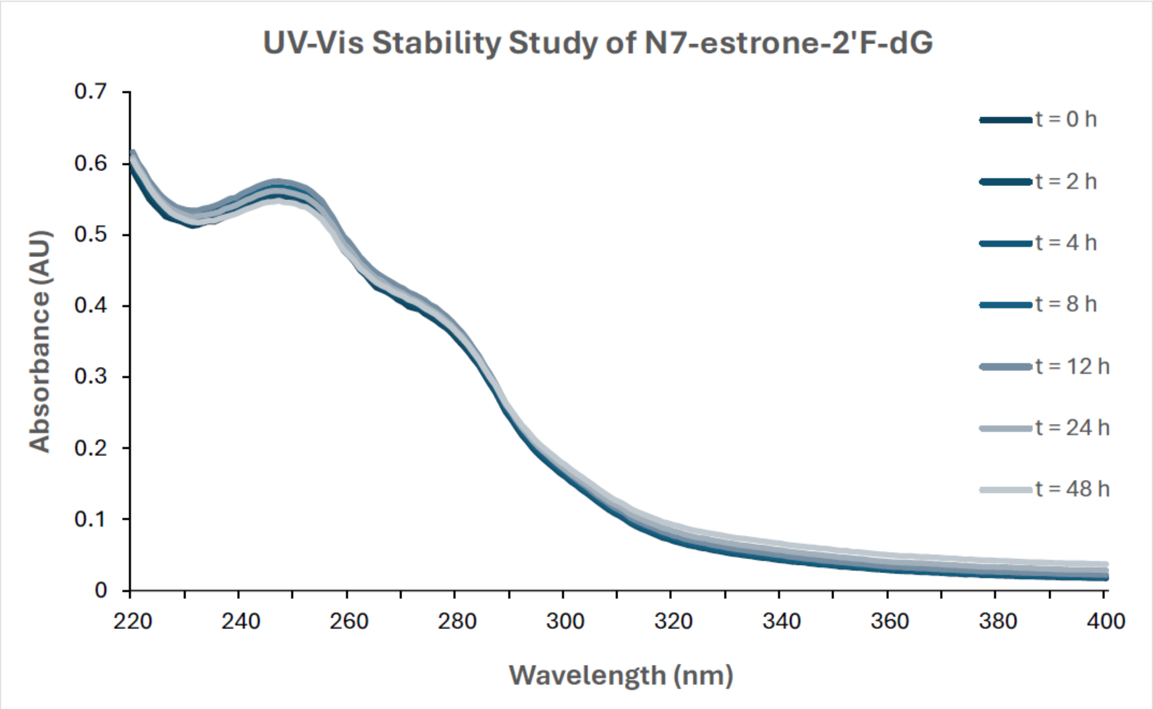

Figure S2. Stacked UV-Vis data of N7-estrone-2′-F-dG incubated at 37°C over a 48 hour period

Table S2. Raw data for Figure S2.

Wavelength (nm)	t = 0 h	t = 2 h	t = 4 h	t = 8 h	t = 12 h	t = 24 h	t = 48 h
220	0.589382	0.597983	0.604602	0.606888	0.616253	0.608698	0.604628
221	0.576139	0.582566	0.589439	0.5916	0.600535	0.595216	0.591234
222	0.562836	0.57127	0.577348	0.580673	0.589806	0.582863	0.57953
223	0.54992	0.557864	0.564187	0.567387	0.576371	0.569189	0.566469
224	0.541801	0.548978	0.554456	0.557293	0.566501	0.559163	0.555629
225	0.533795	0.539727	0.545832	0.548614	0.556636	0.549889	0.546364
226	0.525681	0.532589	0.538576	0.540986	0.54979	0.542896	0.538286
227	0.522379	0.527115	0.533002	0.535623	0.544225	0.536667	0.531474
228	0.520441	0.522613	0.52891	0.532123	0.539179	0.532847	0.526553
229	0.518155	0.520333	0.526204	0.529584	0.536886	0.529816	0.523429
230	0.514858	0.518533	0.523702	0.527901	0.534489	0.527295	0.52015
231	0.512222	0.517389	0.522323	0.525778	0.534375	0.52457	0.517888
232	0.513547	0.518443	0.523367	0.527192	0.534294	0.526118	0.51721
233	0.516217	0.520063	0.525167	0.529335	0.53655	0.527041	0.518048
234	0.518304	0.521657	0.527088	0.531169	0.537974	0.528337	0.519739
235	0.518068	0.523866	0.528888	0.533836	0.540629	0.530218	0.519778
236	0.521434	0.526564	0.532308	0.535528	0.543351	0.532318	0.522546
237	0.524691	0.528928	0.534987	0.539166	0.546888	0.536083	0.5247
238	0.527455	0.531752	0.538524	0.541598	0.550738	0.538456	0.526886
239	0.530744	0.5347	0.540411	0.545725	0.552155	0.541301	0.529114
240	0.53401	0.539176	0.545222	0.549494	0.557136	0.54512	0.53236
241	0.539313	0.54337	0.549381	0.553929	0.560611	0.548694	0.53567
242	0.542823	0.545843	0.552443	0.556365	0.564101	0.551668	0.538397
243	0.544754	0.550143	0.55558	0.560516	0.567773	0.555337	0.541076
244	0.547559	0.551137	0.557666	0.562773	0.57078	0.557793	0.54417
245	0.550763	0.554278	0.561404	0.564843	0.573398	0.560289	0.545638
246	0.553161	0.554902	0.561748	0.567081	0.574889	0.561564	0.546369
247	0.555	0.555965	0.563289	0.567313	0.575364	0.561628	0.54783
248	0.553283	0.554735	0.561913	0.566229	0.574256	0.561261	0.545491
249	0.551947	0.554098	0.560396	0.565521	0.573198	0.559081	0.545128
250	0.549529	0.552208	0.558038	0.563137	0.571069	0.557634	0.543769
251	0.546915	0.549107	0.555062	0.560425	0.568705	0.555052	0.540499
252	0.544624	0.547594	0.552558	0.558047	0.565788	0.552418	0.538949
253	0.538914	0.541614	0.54768	0.553258	0.560827	0.547708	0.533805
254	0.53207	0.535486	0.54202	0.546145	0.554168	0.541794	0.528082
255	0.525044	0.52716	0.533949	0.538247	0.547327	0.534402	0.521408
256	0.513841	0.516545	0.52329	0.52729	0.535153	0.522686	0.511062
257	0.503957	0.506064	0.511984	0.517239	0.524446	0.513418	0.502055
258	0.489987	0.4922	0.498853	0.503055	0.510688	0.49996	0.488863
259	0.477349	0.479308	0.485675	0.489734	0.4971	0.487158	0.478202
260	0.470155	0.470901	0.476512	0.481585	0.489664	0.479731	0.470708
261	0.461308	0.462175	0.468518	0.472957	0.479959	0.470802	0.463145
262	0.450359	0.451241	0.456833	0.461287	0.469217	0.459779	0.452906
263	0.443034	0.444319	0.449672	0.453961	0.460996	0.452738	0.446357
264	0.433985	0.435822	0.44211	0.446354	0.453398	0.445473	0.439533
265	0.425841	0.42951	0.435004	0.440062	0.446893	0.439194	0.433395
266	0.422296	0.424282	0.430527	0.434459	0.44144	0.435489	0.428872
267	0.419156	0.420236	0.426464	0.430178	0.437147	0.4296	0.425317

268	0.414459	0.415242	0.419952	0.4248	0.432924	0.424478	0.420536
269	0.408178	0.410934	0.415969	0.420566	0.427733	0.420597	0.416562
270	0.404743	0.407954	0.412457	0.417762	0.424453	0.417464	0.413624
271	0.398781	0.402577	0.408152	0.412283	0.4192	0.412503	0.408687
272	0.396716	0.399539	0.40452	0.408856	0.4161	0.408972	0.406132
273	0.39451	0.396044	0.401161	0.404901	0.411962	0.405328	0.402283
274	0.388915	0.391581	0.396288	0.400317	0.407744	0.400232	0.396879
275	0.384174	0.385359	0.391513	0.395449	0.401847	0.395272	0.392403
276	0.379127	0.382218	0.387594	0.391137	0.39807	0.390988	0.38811
277	0.373602	0.376101	0.380409	0.384824	0.391462	0.385299	0.38183
278	0.368489	0.369815	0.374742	0.378841	0.385504	0.378674	0.376049
279	0.361305	0.363476	0.368099	0.372205	0.378414	0.372495	0.369775
280	0.352326	0.354781	0.359534	0.363283	0.369598	0.363574	0.361482
281	0.34447	0.346724	0.351525	0.354897	0.361657	0.356412	0.353524
282	0.335709	0.33733	0.341713	0.345585	0.352029	0.34641	0.345102
283	0.325076	0.326662	0.331305	0.33438	0.340336	0.335821	0.334214
284	0.316074	0.316317	0.320131	0.323772	0.329699	0.325705	0.324323
285	0.303657	0.303607	0.307747	0.311116	0.316367	0.312954	0.312098
286	0.290263	0.291004	0.29517	0.297786	0.304519	0.3	0.3
287	0.27892	0.279721	0.283217	0.286764	0.29165	0.289594	0.28965
288	0.265035	0.26527	0.26903	0.272286	0.277127	0.275149	0.276385
289	0.250885	0.251027	0.254053	0.257593	0.262864	0.261267	0.26315
290	0.240673	0.240287	0.244256	0.246216	0.251345	0.25088	0.252736
291	0.229444	0.228801	0.232005	0.234987	0.240488	0.238957	0.242089
292	0.21794	0.218478	0.221474	0.224224	0.229	0.228838	0.232121
293	0.209057	0.209508	0.212249	0.214928	0.219637	0.219737	0.223945
294	0.199347	0.199817	0.202865	0.205401	0.210113	0.210423	0.214684
295	0.191314	0.192191	0.19442	0.197354	0.201925	0.202493	0.206891
296	0.184921	0.184991	0.188227	0.19047	0.195432	0.195759	0.200773
297	0.178756	0.17796	0.180783	0.183579	0.188258	0.18925	0.194124
298	0.172421	0.171485	0.174329	0.176599	0.181164	0.182573	0.188184
299	0.165955	0.164926	0.168003	0.170353	0.174748	0.176461	0.181562
300	0.160795	0.159612	0.161723	0.164052	0.168634	0.170269	0.176506
301	0.15408	0.152939	0.155298	0.157739	0.162229	0.16405	0.170204
302	0.14919	0.14795	0.149724	0.152199	0.156778	0.159037	0.164688
303	0.143167	0.141572	0.143983	0.145982	0.150688	0.153591	0.159692
304	0.138506	0.136214	0.138487	0.140539	0.145255	0.148253	0.154232
305	0.133026	0.130566	0.13289	0.135368	0.138842	0.142715	0.149025
306	0.126301	0.124633	0.12648	0.128683	0.133056	0.136402	0.14312
307	0.120632	0.118788	0.120606	0.123252	0.127444	0.131043	0.138026
308	0.116095	0.1135	0.116064	0.118043	0.122265	0.125874	0.133
309	0.110839	0.108785	0.110525	0.113182	0.117167	0.121529	0.128022
310	0.107043	0.104588	0.106151	0.109024	0.112808	0.117873	0.124435
311	0.10242	0.099996	0.1022	0.104478	0.108776	0.112768	0.120335
312	0.097124	0.094594	0.096358	0.098807	0.102712	0.107663	0.115402
313	0.092636	0.090446	0.092217	0.094462	0.098738	0.103619	0.111526
314	0.089184	0.086806	0.088835	0.091535	0.095102	0.100429	0.108232
315	0.085945	0.083768	0.085573	0.087692	0.091754	0.097183	0.105304
316	0.083654	0.080611	0.082301	0.084654	0.08871	0.09408	0.102607
317	0.079699	0.077322	0.079223	0.081543	0.085692	0.091211	0.099521
318	0.076533	0.073851	0.075661	0.078049	0.082157	0.088126	0.096349

319	0.07465	0.072276	0.074152	0.076325	0.080046	0.086161	0.094763
320	0.071805	0.069359	0.071408	0.073697	0.077344	0.083618	0.092248
321	0.069772	0.06709	0.069174	0.071591	0.075455	0.081931	0.090709
322	0.067329	0.065019	0.06651	0.068954	0.07283	0.078776	0.087873
323	0.065176	0.063139	0.064875	0.067068	0.070801	0.077124	0.085797
324	0.063553	0.061751	0.063152	0.065366	0.069287	0.075323	0.084381
325	0.062011	0.059568	0.061211	0.063589	0.067642	0.073825	0.083072
326	0.060674	0.058748	0.060044	0.062006	0.066239	0.072938	0.081429
327	0.059298	0.056866	0.058261	0.060572	0.06445	0.071195	0.080222
328	0.057803	0.055551	0.057015	0.059424	0.06325	0.06973	0.078796
329	0.056938	0.054257	0.055922	0.05809	0.061782	0.068513	0.077616
330	0.055504	0.052742	0.054168	0.056355	0.060243	0.066971	0.076069
331	0.054306	0.051313	0.052998	0.055064	0.058928	0.065858	0.074797
332	0.052723	0.050439	0.051826	0.05398	0.057771	0.064446	0.073588
333	0.052235	0.049447	0.050814	0.053069	0.056824	0.06339	0.07297
334	0.050898	0.048435	0.049716	0.051588	0.055586	0.062753	0.071899
335	0.050192	0.047181	0.048707	0.050887	0.05471	0.06154	0.070934
336	0.04906	0.046155	0.047316	0.049967	0.053404	0.060635	0.069599
337	0.048132	0.045581	0.046536	0.049023	0.052648	0.059606	0.068736
338	0.047503	0.0446	0.04553	0.047604	0.051258	0.058562	0.067721
339	0.046112	0.043274	0.04472	0.046728	0.050209	0.057388	0.066718
340	0.045611	0.042372	0.043847	0.045733	0.049644	0.056711	0.065881
341	0.043862	0.041349	0.042264	0.044424	0.048071	0.055316	0.064416
342	0.043715	0.040448	0.041794	0.043764	0.047319	0.054291	0.063488
343	0.042589	0.039981	0.041251	0.042985	0.046667	0.053587	0.062879
344	0.041374	0.039341	0.040287	0.042325	0.045215	0.052638	0.06196
345	0.0409	0.038352	0.039104	0.041409	0.044566	0.051452	0.061157
346	0.040239	0.037512	0.038816	0.040858	0.043788	0.051048	0.060494
347	0.039326	0.036649	0.037887	0.03958	0.042917	0.050063	0.05939
348	0.03864	0.036112	0.037115	0.038864	0.041954	0.049548	0.05879
349	0.037958	0.035142	0.036201	0.038205	0.041549	0.048565	0.058164
350	0.037568	0.034578	0.035487	0.037363	0.040602	0.04793	0.057068
351	0.035937	0.033285	0.034488	0.036325	0.039631	0.046577	0.056049
352	0.035839	0.033223	0.034367	0.036277	0.039238	0.046275	0.055928
353	0.034831	0.03231	0.033459	0.035135	0.038382	0.045224	0.054525
354	0.034399	0.032061	0.032992	0.034526	0.037886	0.044742	0.054165
355	0.03363	0.031387	0.032464	0.03409	0.037332	0.044199	0.05353
356	0.032786	0.030485	0.031513	0.03309	0.036359	0.043321	0.052547
357	0.032391	0.030054	0.031158	0.032549	0.035646	0.04249	0.051742
358	0.031727	0.029486	0.030519	0.031871	0.035008	0.041619	0.051035
359	0.031078	0.029001	0.029847	0.031564	0.034371	0.041273	0.050716
360	0.030658	0.028231	0.029376	0.031025	0.03368	0.040442	0.049993
361	0.029695	0.027826	0.028798	0.030362	0.033334	0.039845	0.049302
362	0.029557	0.02741	0.028693	0.029858	0.033058	0.039469	0.048753
363	0.029222	0.02709	0.028223	0.029559	0.03241	0.039371	0.048462
364	0.029318	0.026946	0.027981	0.02946	0.032352	0.038946	0.048409
365	0.028296	0.02596	0.026813	0.028509	0.031574	0.038595	0.04771
366	0.028204	0.025858	0.026756	0.028394	0.03144	0.038384	0.047514
367	0.027976	0.025774	0.026544	0.028144	0.031139	0.038194	0.047187
368	0.027199	0.025041	0.02589	0.027685	0.030426	0.037441	0.046575
369	0.027298	0.024733	0.025721	0.027491	0.030473	0.037412	0.046469

370	0.02633	0.024348	0.024883	0.02678	0.029612	0.036542	0.045706
371	0.026264	0.023829	0.024621	0.026327	0.029284	0.036211	0.045177
372	0.026017	0.023755	0.024812	0.026363	0.029159	0.035931	0.045317
373	0.025435	0.023281	0.024173	0.025697	0.028689	0.035461	0.044407
374	0.024623	0.022507	0.023486	0.024988	0.027999	0.034681	0.043648
375	0.024715	0.022604	0.023523	0.025018	0.028106	0.034615	0.043894
376	0.024537	0.02223	0.023269	0.024397	0.027467	0.034173	0.043257
377	0.023842	0.021739	0.022734	0.024086	0.027023	0.033668	0.042604
378	0.023402	0.021523	0.022332	0.024012	0.026772	0.033318	0.042329
379	0.023533	0.021425	0.022444	0.023932	0.026946	0.0334	0.042297
380	0.023105	0.020806	0.02193	0.023423	0.026081	0.032925	0.04198
381	0.022921	0.020788	0.021965	0.023383	0.026173	0.033091	0.041778
382	0.022777	0.020319	0.021509	0.022734	0.026004	0.032574	0.041356
383	0.02248	0.020329	0.021246	0.022648	0.025584	0.032303	0.041197
384	0.022157	0.019925	0.020856	0.022477	0.025383	0.031889	0.040712
385	0.021918	0.019887	0.020612	0.022193	0.025032	0.031852	0.040346
386	0.021608	0.019703	0.020703	0.021787	0.024901	0.031404	0.040011
387	0.021437	0.019541	0.020356	0.021679	0.024384	0.031205	0.039845
388	0.021111	0.019355	0.020046	0.021538	0.024374	0.030866	0.039652
389	0.020851	0.019019	0.01996	0.021434	0.024307	0.030536	0.039478
390	0.020711	0.018913	0.019703	0.021276	0.024002	0.030444	0.039096
391	0.020282	0.018526	0.019401	0.020708	0.023463	0.029962	0.038767
392	0.020236	0.018427	0.019287	0.020611	0.023447	0.029772	0.038439
393	0.020061	0.018282	0.019047	0.020413	0.023152	0.029535	0.038351
394	0.01991	0.018091	0.019004	0.020452	0.023202	0.029546	0.038193
395	0.01982	0.018022	0.01879	0.020366	0.023099	0.029432	0.037991
396	0.019645	0.017698	0.018678	0.02021	0.022878	0.029359	0.038083
397	0.01951	0.017595	0.018668	0.020173	0.022613	0.029273	0.037878
398	0.019285	0.017426	0.018355	0.020006	0.02253	0.029026	0.037837
399	0.019362	0.01731	0.018175	0.019606	0.022559	0.028899	0.037449
400	0.018798	0.017194	0.017977	0.019402	0.022103	0.028583	0.037201
